# Crystal structure of 3,3′-biisoxazole-5,5′-bis(methyl­ene) dinitrate (BIDN)

**DOI:** 10.1107/S205698901700487X

**Published:** 2017-03-31

**Authors:** Rosario C. Sausa, Rose A. Pesce-Rodriguez, Leah A. Wingard, Pablo E. Guzmán, Jesse J. Sabatini

**Affiliations:** a US Army Research Laboratory, RDRL-WML-B, Aberdeen Proving Ground, MD 21005, USA; b US Army Research Laboratory, RDRL-WML-C, Aberdeen Proving Ground, MD 21005, USA

**Keywords:** crystal structure, 3,3′-bis-isoxazole-5,5′-bis-methyl­ene dinitrate, energetic material, density, FTIR, Raman, and ultraviolet absorption peaks

## Abstract

The mol­ecular structure of the title energetic compound, C_8_H_6_N_4_O_8_, is composed of two planar isoxazole rings and two near planar alkyl-nitrate groups (r.m.s deviation = 0.006 Å). In the crystal, the mol­ecule sits on an inversion center, thus *Z*′ = 0.5. The dihedral angle between the isoxazole ring and the nitrate group is 69.58 (8)°. van der Waals contacts dominate the inter­molecular inter­actions. Inversion-related rings are in close slip-stacked proximity, with an inter­planar separation of 3.101 (3) Å [centroid–centroid distance = 3.701 (3) Å]. The measured and calculated densities are in good agreement (1.585 *versus* 1.610 Mg m^−3^).

## Abstract 2

The crystal structure and packing of the energetic compound 3,3′-bis-isoxazole-5,5′-bis-methyl­ene dinitrate is reported. Major FTIR, Raman, UV absorption peaks, as well as experimental and calculated density are reported.

## Chemical context   

Isoxazole compounds have attracted much inter­est in recent years because of their potential usefulness in medicine, agriculture, and in the field of energetic materials (Galenko *et al.*, 2015 ▸; Wingard *et al.*, 2017 ▸). The title compound is an isoxazole-based energetic material that has been synthesized recently in our laboratory. It has potential use as a tri­nitro­toluene replacement in melt-castable and Composition B formulations, and as an energetic plasticizing ingredient in nitro­cellulose-based propellant formulations. The compound is composed of two heterocyclic isoxazole rings, each bonded to an alkyl nitric ester group. The heterocyclic base has non-bonded electron lone pairs which can exhibit Lewis-base behavior towards electrophilic materials such as nitro­cellulose, whereas the alkyl nitric esters provide miscibility and compatibility with commonly used energetic plasticizers.
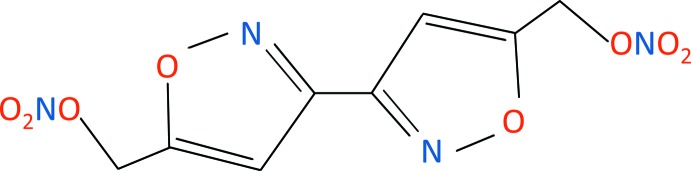


## Structural commentary   

**Figure 1:**
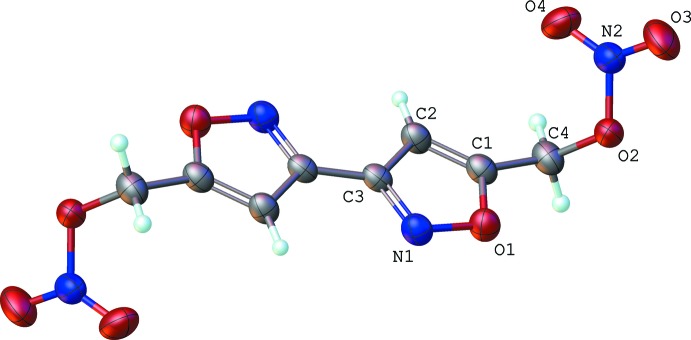
Mol­ecular conformation and atom-numbering scheme. Non-labeled atoms are generated by inversion (−*x*, 1 − *x*, 1 − *z*). Non-hydrogen atoms are shown as 50% probability displacement ellipsoids.

The mol­ecule (see Fig. 1 ▸) consists of two isoxazole rings bonded to two alkyl nitric ester groups. There are no unusual bond lengths or angles. The rings are planar (r.m.s. deviation = 0.0003 Å), and adopt a co-planar *trans* geometry, perhaps to minimize lone-pair inter­actions of the nitro­gen atoms, similar to 3,3′-bis­oxazole and 5,5′-diphenyl-3,3′-bis­oxazole (Cannas & Marongiu, 1968 ▸; van der Peet *et al.*, 2013 ▸). Atom C4 is co-planar with the ring [deviation = 0.062 (3) Å]. Similarly, atoms C4/O2/N2/O3/O4 adopt a near planar conformation (r.m.s deviation = 0.006 Å). The dihedral angle between the isoxazole ring and the nitrate group is 69.58 (9)°.

## Supra­molecular features   

**Figure 2:**
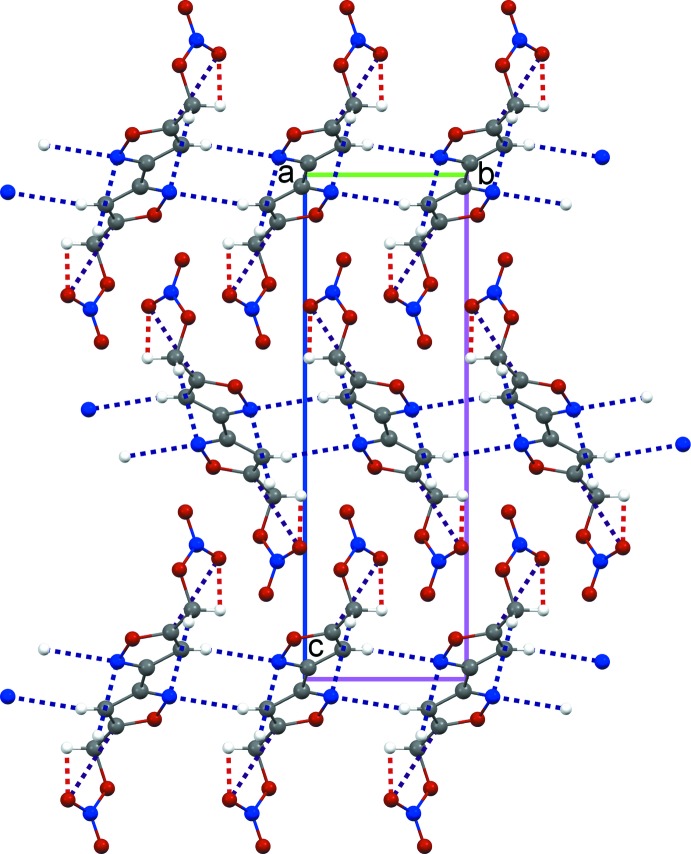
Crystal packing viewed along the *a* axis. Dashed lines represent contacts between atoms N1⋯H2, N11⋯H4*A*, and C11⋯O4 (blue) and O41⋯H4*B* (red).

Figs. 2 ▸ and 3 ▸ show the packing of the title compound along the *a* and *b* axes, respectively. Bifurcated contacts between the N1 and H atoms of adjacent mol­ecules [N1⋯H4*A* ^i^ = 2.704 (4) Å and N1⋯H2^ii^ = 2.656 (4) Å); symmetry codes: (i) 1 − *x*, 1 − *y*, 1 − *z*; (ii) *x*, *y* − 1, *z*] dominate the inter­molecular inter­actions. Inversion-related (1 − *x*, 1 − *y*, 1 − *z*) isoxazole rings are in close slip-stacked proximity, with an inter­planar separation of 3.101 (3) Å [ring centroid–centroid distance = 3.701 (3) Å].

## Database survey   

An open literature search, as well as a search of the Cambridge Structural Database (Groom *et al.*, 2016 ▸) and the Crystallography Open Database (Gražulis *et al.*, 2009 ▸) yielded many hits for bis-isoxazole-containing compounds and several on 3,3′ and 5,5′ bis-isoxazole-based compounds, the most pertinent studies relating to the title compound being the crystal structures of 3,3′-bis­oxazole (Cannas & Marongiu, 1968 ▸; CCDC 1111317, BIOXZL) and 5,5′-diphenyl-3,3′-bis­oxazole (van der Peet *et al.*, 2013 ▸; CCDC 935274). In these compounds, the rings also adopt planar *trans* conformations, similar to that observed in the title compound.

## Synthesis and crystallization   

The synthesis of the title compound has been reported recently (Wingard *et al.*, 2017 ▸). Briefly, a solution of sodium bicarbonate was added to a mixture of di­chloro­glyoxime (0.191 mol), propargyl alcohol (0.956 mol), and 1.9 L of methanol to produce the inter­mediate compound 5,5′-di­hydroxy­methyl-3,3′-bis-isoxazole (75% yield). Then, this compound (0.120 mol) was added portionwise over ten minutes to 90% nitric acid (150 ml) placed in a 250 ml round-bottom flask equipped with a stir bar, and cooled in an ice–water bath. No exotherm was observed during the addition. The reaction mixture was stirred for four hours while the water–ice bath was warmed to room temperature. The reaction mixture was poured onto ice, resulting in the formation of a white precipitate, which was collected by Büchner filtration and dried, giving the title compound (92% yield). Slow solvent evaporation of a solution in aceto­nitrile yielded suitable single crystals for the X-ray diffraction experiments at room temperature. Based on the cell dimensions and mol­ecular weight, the calculated crystal density of 1.609 Mg m^−3^ at 297 K is in excellent agreement with the value of 1.585 Mg m^−3^ measured using a pycnometer at room temperature.

Spectroscopic data: FTIR (Nicolet iS50, attenuated total reflectance, cm^−1^): 3144 (*w*), 3032 (*w*), 2923 (*w*), 1643 (*m*), 1605 (*m*), 1421 (*m*), 1359 (*m*), 1351(*m*), 1278 (*s*), 1259 (*m*), 1209 (*m*), 1075 (*m*), 1021 (*w*), 955 (*m*), 926 (*s*), 912 (*s*), 845 (*s*), 824 (*s*), 753 (*s*), 649 (*m*), 582 (*m*). Raman (Nicolet iS50, 1064 nm; cm^−1^): 3143 (*m*), 3027 (*w*), 2977 (*m*), 2855.59 (*w*), 1621 (*w*), 1552 (*s*), 1476 (*m*), 1422 (*w*), 1354 (*w*), 1299 (*w*) 1279 (*w*), 1146 (*w*), 1020 (*w*) 960 (*m*), 922 (*w*), 847 (*m*), 728 (*w*), 667 (*w*), 645 (*w*), 585 (*m*), 489 (*m*), 449 (*w*), 381 (*w*), 373 (*w*), 249 (*w*), 218 (*w*), 161.70 (*w*). UV (aceto­nitrile solvent, nm): 220 nm (max).

## Refinement   

Crystal data, data collection and structure refinement details are summarized in Table 1 ▸. The hydrogen atoms were refined using a riding model with C—H = 0.93 or 0.97 Å and *U*_iso_(H) = 1.2*U*_eq_(C).

## Supplementary Material

Crystal structure: contains datablock(s) I. DOI: 10.1107/S205698901700487X/pk2599sup1.cif

CCDC reference: 1540757

Additional supporting information:  crystallographic information; 3D view; checkCIF report

## Figures and Tables

**Figure 3:**
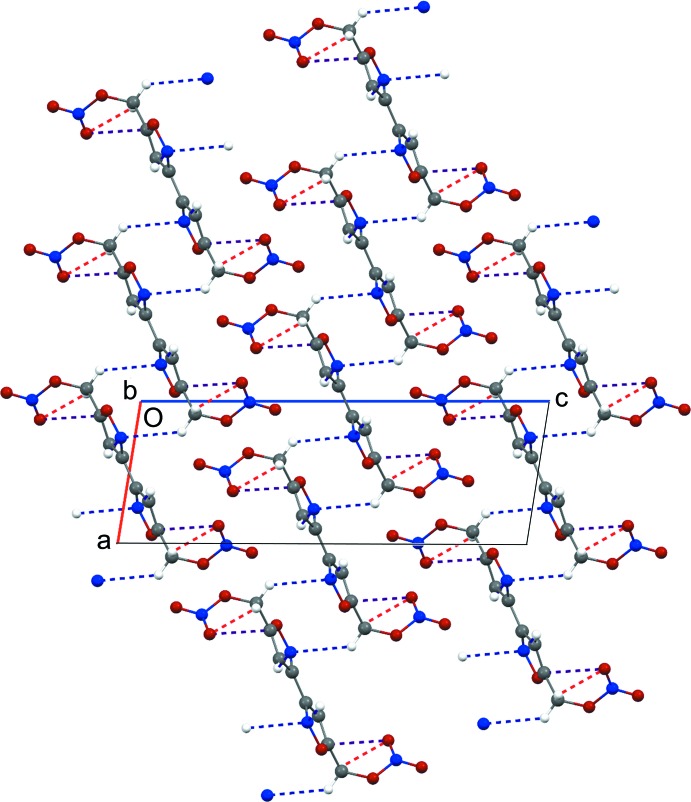
Crystal packing viewed along the *b* axis. Dashed lines represent contacts between atoms N1⋯H4*A* and C11⋯O4 (blue), and O4⋯H4*B* (red).

**Table 1:** Experimental details

Crystal data	
Chemical formula	C_8_H_6_N_4_O_8_
*M*_r_	286.17
Crystal system, space group	Monoclinic, *P*2_1_/*n*
Temperature (K)	297
*a*, *b*, *c* (Å)	6.1917 (5), 5.5299 (5), 17.4769 (12)
β (°)	99.233 (7)
*V* (Å^3^)	590.65 (8)
*Z*	2
Radiation type	Mo *K*α
μ (mm^−1^)	0.15
Crystal size (mm)	0.4 × 0.2 × 0.1

Data collection	
Diffractometer	Agilent SuperNova, Dualflex, EosS2
Absorption correction	Multi-scan (SCALE3 ABSPACK in *CrysAlis PRO*; Rigaku OD, 2015 ▸; Bourhis *et al.*, 2015 ▸)
*T*_min_, *T*_max_	0.678, 1.000
No. of measured, independent and observed [*I* > 2σ(*I*)] reflections	4487, 1079, 903
*R*_int_	0.027
(sin θ/λ)_max_ (Å^−1^)	0.602

Refinement	
*R*[*F* ^2^ > 2σ(*F* ^2^)], *wR*(*F* ^2^), *S*	0.041, 0.105, 1.06
No. of reflections	1079
No. of parameters	92
H-atom treatment	H-atom parameters constrained
Δρ_max_, Δρ_min_ (e Å^−3^)	0.19, −0.18

Computer programs: *CrysAlis PRO* (Rigaku OD, 2015 ▸), *SHELXT* (Sheldrick, 2015 ▸ *a*), *SHELXL* (Sheldrick, 2015 ▸ *b*), *OLEX2* (Dolomanov *et al.*, 2009 ▸) and *Mercury* (Macrae *et al.*, 2008 ▸).

## supplementary crystallographic information

### Crystal data

**Table:**

C_8_H_6_N_4_O_8_	*F*(000) = 292
*M**_r_* = 286.17	*D*_x_ = 1.609 Mg m^−^^3^
Monoclinic, *P*2_1_/*n*	Mo *K*α radiation, λ = 0.71073 Å
*a* = 6.1917 (5) Å	Cell parameters from 1878 reflections
*b* = 5.5299 (5) Å	θ = 2.4–25.2°
*c* = 17.4769 (12) Å	µ = 0.15 mm^−^^1^
β = 99.233 (7)°	*T* = 297 K
*V* = 590.65 (8) Å^3^	Irregular, colourless
*Z* = 2	0.4 × 0.2 × 0.1 mm

### Data collection

**Table:**

SuperNova, Dualflex, EosS2 diffractometer	1079 independent reflections
Radiation source: fine-focus sealed X-ray tube, Enhance (Mo) X-ray Source	903 reflections with *I* > 2σ(*I*)
Graphite monochromator	*R*_int_ = 0.027
Detector resolution: 8.0945 pixels mm^-1^	θ_max_ = 25.3°, θ_min_ = 2.4°
ω scans	*h* = −7→7
Absorption correction: multi-scan (SCALE3 ABSPACK in CrysAlisPro; Rigaku OD, 2015; Bourhis *et al.*, 2015)	*k* = −6→5
*T*_min_ = 0.678, *T*_max_ = 1.000	*l* = −21→21
4487 measured reflections	

### Refinement

**Table:**

Refinement on *F*^2^	Hydrogen site location: inferred from neighbouring sites
Least-squares matrix: full	H-atom parameters constrained
*R*[*F*^2^ > 2σ(*F*^2^)] = 0.041	*w* = 1/[σ^2^(*F*_o_^2^) + (0.045*P*)^2^ + 0.168*P*] where *P* = (*F*_o_^2^ + 2*F*_c_^2^)/3
*wR*(*F*^2^) = 0.105	(Δ/σ)_max_ < 0.001
*S* = 1.06	Δρ_max_ = 0.19 e Å^−^^3^
1079 reflections	Δρ_min_ = −0.18 e Å^−^^3^
92 parameters	Extinction correction: SHELXL-2016/4 (Sheldrick 2015b), Fc^*^=kFc[1+0.001xFc^2^λ^3^/sin(2θ)]^-1/4^
0 restraints	Extinction coefficient: 0.077 (8)
Primary atom site location: dual	

### Special details

**Table:**

Geometry. All esds (except the esd in the dihedral angle between two l.s. planes) are estimated using the full covariance matrix. The cell esds are taken into account individually in the estimation of esds in distances, angles and torsion angles; correlations between esds in cell parameters are only used when they are defined by crystal symmetry. An approximate (isotropic) treatment of cell esds is used for estimating esds involving l.s. planes.

### Fractional atomic coordinates and isotropic or equivalent isotropic displacement parameters (Å^2^)

**Table:**

	*x*	*y*	*z*	*U*_iso_*/*U*_eq_	
C4	0.5869 (4)	0.8027 (4)	0.63740 (11)	0.0621 (6)	
H4A	0.714559	0.780169	0.612468	0.075*	
H4B	0.553186	0.974123	0.636451	0.075*	
C3	0.1082 (3)	0.5201 (3)	0.52299 (9)	0.0446 (5)	
C1	0.3989 (3)	0.6693 (3)	0.59298 (10)	0.0513 (5)	
C2	0.1987 (3)	0.7337 (3)	0.55899 (10)	0.0512 (5)	
H2	0.133409	0.885170	0.559012	0.061*	
N1	0.2447 (3)	0.3392 (3)	0.53455 (9)	0.0558 (5)	
N2	0.5082 (3)	0.8284 (4)	0.76635 (11)	0.0660 (5)	
O2	0.6372 (2)	0.7230 (3)	0.71689 (8)	0.0610 (5)	
O1	0.4340 (2)	0.4322 (2)	0.57971 (8)	0.0603 (4)	
O4	0.3714 (3)	0.9669 (4)	0.73937 (12)	0.0920 (6)	
O3	0.5589 (3)	0.7599 (4)	0.83151 (10)	0.1017 (7)	

### Atomic displacement parameters (Å^2^)

**Table:**

	*U*^11^	*U*^22^	*U*^33^	*U*^12^	*U*^13^	*U*^23^
C4	0.0688 (13)	0.0653 (15)	0.0515 (11)	−0.0108 (11)	0.0070 (10)	−0.0053 (9)
C3	0.0621 (11)	0.0348 (9)	0.0377 (9)	−0.0046 (8)	0.0103 (7)	0.0013 (7)
C1	0.0674 (13)	0.0421 (11)	0.0444 (10)	−0.0060 (9)	0.0088 (9)	−0.0012 (8)
C2	0.0678 (13)	0.0361 (10)	0.0478 (10)	−0.0027 (9)	0.0037 (9)	−0.0010 (8)
N1	0.0665 (11)	0.0421 (10)	0.0571 (9)	−0.0043 (8)	0.0046 (8)	−0.0043 (7)
N2	0.0596 (11)	0.0725 (13)	0.0640 (12)	−0.0003 (10)	0.0042 (9)	−0.0189 (9)
O2	0.0597 (8)	0.0670 (10)	0.0536 (8)	0.0124 (7)	0.0009 (6)	−0.0115 (7)
O1	0.0649 (9)	0.0490 (9)	0.0637 (8)	0.0005 (7)	0.0002 (7)	−0.0044 (6)
O4	0.0747 (11)	0.0866 (13)	0.1133 (14)	0.0257 (10)	0.0113 (10)	−0.0237 (11)
O3	0.1055 (14)	0.144 (2)	0.0538 (10)	−0.0022 (13)	0.0082 (9)	−0.0135 (11)

### Geometric parameters (Å, º)

**Table:**

C4—H4A	0.9700	C1—C2	1.334 (3)
C4—H4B	0.9700	C1—O1	1.355 (2)
C4—C1	1.487 (3)	C2—H2	0.9300
C4—O2	1.443 (2)	N1—O1	1.402 (2)
C3—C3^i^	1.465 (4)	N2—O2	1.395 (2)
C3—C2	1.411 (2)	N2—O4	1.182 (2)
C3—N1	1.304 (2)	N2—O3	1.193 (2)
			
H4A—C4—H4B	107.9	O1—C1—C4	115.83 (18)
C1—C4—H4A	109.1	C3—C2—H2	127.8
C1—C4—H4B	109.1	C1—C2—C3	104.42 (17)
O2—C4—H4A	109.1	C1—C2—H2	127.8
O2—C4—H4B	109.1	C3—N1—O1	105.57 (15)
O2—C4—C1	112.39 (17)	O4—N2—O2	117.91 (19)
C2—C3—C3^i^	129.4 (2)	O4—N2—O3	130.4 (2)
N1—C3—C3^i^	118.8 (2)	O3—N2—O2	111.70 (19)
N1—C3—C2	111.83 (16)	N2—O2—C4	114.35 (16)
C2—C1—C4	133.91 (19)	C1—O1—N1	107.99 (14)
C2—C1—O1	110.19 (16)		
			
C4—C1—C2—C3	176.7 (2)	C2—C1—O1—N1	−0.1 (2)
C4—C1—O1—N1	−177.37 (15)	N1—C3—C2—C1	0.0 (2)
C3^i^—C3—C2—C1	179.9 (2)	O2—C4—C1—C2	115.6 (2)
C3^i^—C3—N1—O1	−179.97 (18)	O2—C4—C1—O1	−67.9 (2)
C3—N1—O1—C1	0.08 (19)	O1—C1—C2—C3	0.0 (2)
C1—C4—O2—N2	−82.9 (2)	O4—N2—O2—C4	1.0 (3)
C2—C3—N1—O1	−0.1 (2)	O3—N2—O2—C4	−178.72 (19)

Symmetry code: (i) −*x*, −*y*+1, −*z*+1.
